# Mechanism and application of copper-based nanomedicines in activating tumor immunity through oxidative stress modulation

**DOI:** 10.3389/fphar.2025.1646890

**Published:** 2025-07-11

**Authors:** Cong Xia, Zirun Luo, Zhen Feng, Qianshi Zhang, Chenglai Xia

**Affiliations:** ^1^ Department of Gastrointestinal Surgery, The Second Hospital of Dalian Medical University, Dalian, China; ^2^ Department of Breast, Thyroid and Head-Neck Surgery, The Third Xiangya Hospital of Central South University, Changsha, China; ^3^ Scientific Research Center, Foshan Women and Children Hospital Affiliated with Guangdong Medical University, Foshan, China

**Keywords:** copper, nanomedicine, oxidative stress, tumor immunotherapy, regulated cell death (RCD)

## Abstract

Immunotherapy stands as a powerful weapon against tumors. However, tumor cells evade recognition and attack by the immune system through various mechanisms, achieving immune escape and exhibiting resistance to immunotherapy. Metalloimmunotherapy, as an emerging paradigm for immunotherapy, offers the potential to effectively overcome the limitations of current tumor immunotherapies. Nevertheless, developing highly efficient and specific metal-based agents for regulating the tumor immune system remains a significant challenge. The modulation of oxidative stress in the tumor microenvironment (TME) by metals presents novel breakthroughs for metalloimmunotherapy, particularly in enhancing immune responses, optimizing immune cell function, and reprogramming the immunosuppressive TME. Copper, a transition metal closely associated with tumor development, acts as an immune activator to enhance immune responses through oxidative stress. Benefiting from advances in nanomaterials, copper-based nanomedicines have demonstrated significant potential in improving the efficacy of cancer immunotherapy by modulating oxidative stress via Fenton-like reactions and enzymatic catalytic activities. Therefore, summarizing recent advances in copper-based nanomedicine activating tumor immunity through oxidative stress modulation provides new insights and drives progress for metalloimmunology. This review outlines strategies utilizing oxidative stress modulated by copper-based nanomedicines to induce or enhance immunotherapy through multiple forms of regulated cell death (RCD), drug co-delivery approaches, and versatile combination therapies. Finally, we discuss current challenges and offer perspectives on copper-based nanomedicines in tumor immunotherapy. Our review aims to elucidate the potential of copper-based nanomedicines in tumor immunology, providing insights for the future development of tumor immunotherapies based on metal and redox biology.

## 1 Introduction

Cancer has emerged as a major public health challenge. In cancer treatment, conventional therapies like chemotherapy and radiotherapy are limited by their inefficiency in achieving satisfactory clinical outcomes due to poor tumor targeting and inherent therapeutic resistance (Yang et al., 2024). Immunotherapies, such as immune checkpoint blockade and adoptive cell therapy, have enabled durable remission and long-term survival in previously untreatable patients (Sangro et al., 2021; Xia et al., 2023). Nevertheless, its efficacy varies considerably, with reactivation of immune responses occurring in only a relatively small subset of cancer patients. The dynamic interplay between tumors and the immune system is highly complex, with interactions between tumor and immune evolving throughout tumor progression to modulate immune surveillance and escape (Galassi et al., 2024). The emergence of intrinsic and acquired resistance to immunotherapy has diminished its promise, highlighting significant gaps in our understanding of tumor evolution under immunotherapy pressure. Building on this understanding, the development of novel targets, personalized tumor vaccines, and immune modulators targeting the tumor microenvironment holds promise for overcoming the current limitations of immunotherapy. As insights of immune discontinuity theory and TIME heterogeneity deepen, cutting-edge studies have demonstrated the crucial roles of non-specific chemical entities, such as oxygen, carbon dioxide, lactate, and metal ions, in tumor immunology (Wang et al., 2020; Mai et al., 2025). Transition metals, being of fundamental importance to biocatalysis and metalloallostery in biological processes, are intimately linked with tumor development (Tsang et al., 2021; Bernal et al., 2025). The crucial influences of metal ions on immunity include the cyclic guanosine monophosphate-adenosine monophosphate synthase-stimulator of interferon genes (cGAS-STING) signaling pathway (Zn^2+^ and Mn^2+^) and pathogen-host interactions (Zn^2+^, Fe^2+^/^3+^, Mn^2+^, and Cu^2+^) (Li et al., 2022). The critical role of metal ions in tumor immunomodulation, stemming from their unique electronic structures and Fenton reactivity, has given rise to the concept of metalloimmunotherapy. However, developing metal-based agents that combine high efficiency and specificity for regulating the tumor immune system remains a daunting challenge.

Copper, the third most abundant trace metal in humans after zinc and iron, is essential for immune system regulation (Ge et al., 2022). Elevated serum and tissue copper levels occur in multiple cancers, where it exerts immunomodulatory effects through oxidative stress modulation. Nanostructured therapeutic systems established on copper-based agents have garnered significant attention, demonstrating broad applicability in synergistic cancer immunotherapy via amplified oxidative stress (Xie J. et al., 2023). Compared to other metal-based nanomedicines, copper-based nanomedicines induce broader forms of regulated cell death (RCD) in tumor cells and elicit stronger immune activation. They also produce synergistic effects with other therapies, making them promising and versatile tools for developing effective cancer immunotherapies. Due to weak catalytic capacity for Fenton-like reactions and poor ability to cycle between valence states, the anti-tumor immune response generated by manganese-based and zinc-based nanomedicines alone is limited (Sun et al., 2025). Traditional copper-based nanomedicines exhibit superior Fenton reaction kinetics compared to iron-based counterparts (Cu^2+^ rate constant: 460 M^-1^ s^-1^; Cu^+^ rate constant: 1 × 10^4^ M^-1^ s^-1^), with broader pH applicability (Zhou et al., 2021). As one of copper’s primary oxidation states, Cu^2+^ can be reduced to Cu^+^ by glutathione (GSH). After GSH depletion, the generated Cu^+^ catalyzes H_2_O_2_ decomposition to produce cytotoxic •OH with 160-fold higher Fenton activity than Fe^2+^. Furthermore, copper nanomaterials possess multiple enzyme-like activities, including peroxidase-like (POD), oxidase-like (OXD), superoxide dismutase-like (SOD), catalase-like (CAT), glutathione oxidase-like (GSHOx), and glutathione peroxidase-like (GPx) activity. With their potent single enzyme-like activity or cascades composed of multiple enzyme-like activities, copper-based nanomedicines serve as ideal nanocatalysts for generating endogenous reactive oxygen species (ROS) and remodeling the tumor microenvironment (TME) (Hao et al., 2021). For immunosuppressive TIME, copper-based nanomedicines exert pivotal effects. By disrupting tumor copper and redox homeostasis, copper-based nanomedicines extensively crosstalk with virtually all RCD pathways, ultimately inducing damage-associated molecular pattern (DAMP) release and immunogenic cell death (ICD). With their intrinsic redox properties, copper-based nanomedicines modulate the metabolism of small molecules in TIME to counteract immunosuppression. More importantly, the effect brought by copper-based nanomedicines stimulates immune cells to activate both innate and adaptive immune responses. These findings underscore the multiple roles of copper-based nanomedicines in modulating oxidative stress within complex TIME. Therefore, elucidating copper-dependent redox targets and molecular mechanisms provides crucial insights for developing strategies that exploit copper’s tumor susceptibility and pharmacologically modulate TIME copper/redox homeostasis to enhance immunotherapy efficacy.

This review aims to outline the multifaceted roles of copper-based nanomedicines in tumor immune activation through oxidative stress regulation, including the intricate crosstalk between oxidative stress modulation and various RCD pathways in restoring tumor immunogenicity, the stimulation of immune cells by oxidative stress modulation, and the impact on the immunosuppressive TIME. Finally, we systematically summarize co-delivery strategies and immunotherapies based on engineered copper-based nanomedicines, and discuss current challenges and future directions in leveraging this powerful tool to advance cancer immunotherapy.

## 2 Mechanisms in activating anti-tumor immunity

**FIGURE 1:**
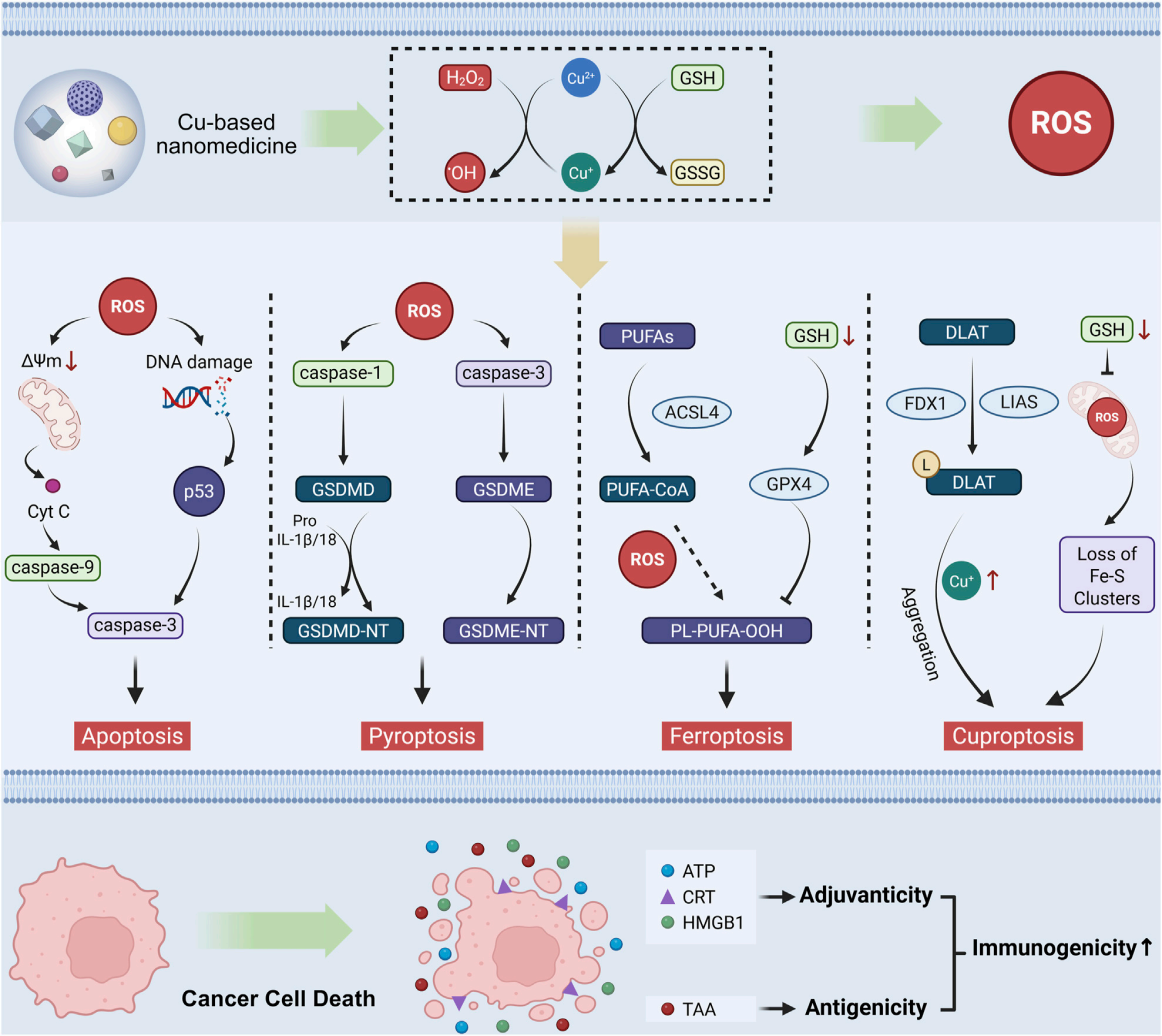
Mechanisms of copper-based nanomedicines in activating tumor immunity through oxidative Stress modulation. Copper-based nanomedicine enhances the immunogenicity of tumor cells by inducing multiple forms of RCD, including apoptosis, pyroptosis, ferroptosis, and cuproptosis. Subsequently, the dying cells release DAMPs to exert an adjuvant effect for immune activation, along with TAAs to elevate tumor antigenicity. These mechanisms act synergistically to collectively restore tumor immunogenicity.

The low immunogenicity of tumors is a main factor in establishing an immunosuppressive TIME. Enhancing tumor immunogenicity enables the immune system to precisely distinguish cancer cells from normal cells, thereby achieving accurate and efficient tumor immunotherapy (Guan et al., 2023). Immunogenicity in cell death comprises antigenicity (derived from tumor-associated antigens (TAAs) and tumor neoantigens) and adjuvanticity (originating from microbe-associated or damage-associated molecular patterns). Crosstalk between different forms of RCD and ICD triggers DAMPs and TAAs release, activating durable tumor-specific immune responses (Xi et al., 2024). The role of oxidative stress modulated by copper-based nanomedicine in tumor immunotherapy primarily arises from ROS, either promoting DAMP release or functioning as intrinsic DAMPs, ultimately engaging the classical cancer-immunity cycle to trigger antitumor immunity. Following the uptake of DAMPs released by dying cancer cells, dendritic cells (DCs) upregulate the expression of CD86 and CD80. These matured DCs migrate to draining lymph nodes, enhance antigen presentation, and induce infiltration of CD8^+^ cytotoxic T lymphocytes (CTLs) and CD4^+^ helper T cells into the TIME, ultimately initiating anti-tumor immunity (Wang et al., 2021; Zhan et al., 2024). Notably, recent studies highlight that copper-based nanomedicines exhibit complex crosstalk across multiple RCD modalities, including apoptosis, pyroptosis, ferroptosis, and cuproptosis, culminating in DAMP release and restoration of tumor immunogenicity (Figure 1) (Gao and Zhang, 2023; Guo et al., 2025). Therefore, understanding these molecular mechanisms will guide future research on tumor immune activation strategies, especially by improving adjuvanticity.

### 2.1 Induction of tumor cell apoptosis

Apoptosis is conventionally regarded as an immunologically silent RCD mechanism. However, therapeutic strategies like ROS generation can restore tumor immunogenicity through apoptosis induction. When phagocytic capacity is inadequate, apoptotic tumor cells expose and release immune-triggering molecules (e.g., DAMPs, chemokines, cytokines) to activate antitumor immunity. As a redox-active metal, copper induces apoptosis via ROS, DNA damage, and proteasome inhibition, modulating tumor apoptotic sensitivity and immune responses (Xue et al., 2023; Bernal et al., 2025).

The copper (Ⅱ) complex of salicylate phenanthroline induces colorectal cancer cell apoptosis through ROS while enhancing ICD via endoplasmic reticulum (ER) stress and amplified ROS generation (Liu Z. et al., 2023; Chen et al., 2024). Disulfiram (DSF), a copper chelator, synergizes with copper by facilitating intracellular copper transport. DSF/Cu-induced apoptosis transforms dying cancer cells into endogenous vaccines via DAMP release. DSF/Cu sustains high ROS levels, causing persistent oxidative stress, DNA damage, and innate immune activation by downstream apoptotic pathways. DSF/Cu treatment induces apoptosis in breast cancer cells and releases DAMPs, including calreticulin (CRT), high mobility group box 1 (HMGB1), heat shock protein (HSP) 70, and HSP 90 (Guo et al., 2024). ER critically mediates ROS accumulation for immunogenicity restoration. DSF/Cu induces apoptosis and ER stress, upregulating CHOP, Xbp1s, p-EIF2α, and p-IRE1 while enhancing DAMPs release in oral squamous cell carcinoma (Zhao Y. et al., 2024).

By catalyzing •OH generation via single-atom-like form, CuCH-NCs combined with DSF elevate cleaved caspase-3 expression and immunogenicity through CRT/HMGB1/adenosine triphosphate (ATP) upregulation, achieving efficient primary/metastatic tumor suppression in triple-negative breast cancer with anti-programmed death-ligand 1 (PD-L1) therapy (Li et al., 2023). FeCu-DA enables cascade reactions via CAT-like, GSHOx-like, and POD-like activities, continuously generating •OH while depleting GSH. With near-infrared (NIR) irradiation, FeCu-DA downregulates Bcl-2, upregulates Bax, and activates caspase-3 to induce apoptosis, triggering abscopal immunity via DAMP release (Ning et al., 2025). Vitamin K3-loaded copper zinc ferrite nanoparticles (NPs) (Vk3@Si@CuZnIONPs) activate the mitochondrial caspase pathway via ROS, upregulating caspase-3 while eliciting dual immunogenic effects through ROS/heat-mediated HSP70/HSP90 upregulation (Chauhan et al., 2023). GCT NPs promote oxidative stress by GSH depletion and ROS production, inducing mitochondrial dysfunction through Bax/p53/PTEN upregulation and Bcl-2 downregulation. Parallel ER stress enhances immunotherapy by DAMPs release (Guo et al., 2022). Hollow Cu_2_MoS_4_ NPs elevate caspase-3, cleaved caspase-3, and caspase-8 expression while upregulating p53 and downregulating Bcl-2, inducing apoptosis and CRT exposure (Yao et al., 2024).

### 2.2 Induction of tumor cell pyroptosis

Pyroptosis is a gasdermin (GSDM)-mediated RCD characterized by membrane pore formation, cellular swelling, and blebbing. Cleavage of conserved domains in GSDM family members (e.g., GSDMD, GSDME) triggers pyroptosis. Typically, activated caspase-1/4/5/11 cleaves GSDMD, releasing its N-terminal domain to form plasma membrane pores. Under specific conditions, apoptosis-dependent caspase-8 directly cleaves GSDMD. GSDME can be cleaved by caspase-3/8, converting non-inflammatory apoptosis to pyroptosis. This process causes cytoplasmic swelling, membrane rupture, and release of cytoplasmic contents, including DAMPs and pro-inflammatory cytokines, making pyroptosis a critical RCD modality in tumor immune (Tan et al., 2021).

Copper-based nanomedicines induce pyroptosis by promoting ROS production to trigger ER stress and upregulate pyroptosis-related genes. Copper-tannic acid nanoneedle (CuTA) catalyzes a ROS storm in tumor cells with SOD-, CAT-, GSH-Px-, and POD-like activities, causing mitochondrial damage and cytochrome c release to initiate inflammation. These effects activate the inflammasome and caspase-3-mediated GSDME cleavage while releasing DAMPs, effectively activating anti-tumor immunity (Wang et al., 2024a). M-Cu-T depletes GSH by Cu^2+^ to promote ROS accumulation, activating the caspase-3/GSDME cascade and triggering immunogenic pyroptosis via DAMP release (Wang et al., 2023). CP@Gel, a hydrogel-encapsulated self-catalytic nanoplatform, generates ROS via Cu^2+^ catalysis to activate caspase-3/GSDME. This initiates pyroptosis with concurrent release of ATP, HMGB1, interleukin-1β (IL-1β), and interferon-γ (IFN-γ), enhancing tumor immunogenicity and reversing immunosuppressive TIME (Rao et al., 2025). In addition to GSDME-mediated pyroptosis, the Cu-TBB nanomedicine releases Cu^+^ to generate ROS with O_2_ as a substrate in tumor cells, activating caspase-1-mediated classical pyroptosis via Gasdermin-D cleavage and promoting release of IL-1β, IL-18, and DAMPs (Zhang Y. et al., 2023).

### 2.3 Induction of tumor cell ferroptosis

Ferroptosis is an iron-dependent RCD driven by phospholipid peroxidation and ROS overload, primarily regulated by iron homeostasis and oxidative stress. Iron excess induces ROS generation and activates iron-containing enzymes, leading to subsequent lipid peroxidation. The glutathione peroxidase 4 (GPX4)-dependent system, which relies on solute carrier family 7 member 11 (SLC7A11)-facilitated extracellular cystine uptake, constitutes the basic defense against ferroptosis by utilizing GSH to directly eliminate lipid peroxides (Lei et al., 2024). Dying cells contribute to anti-tumor immunity activation by releasing DAMPs, which modulate the activation of immune cells.

Compared to iron ions, copper-based nanomedicines induce tumor ferroptosis and potent antitumor immune responses through enhanced Fenton-like reactivity and mitochondrial damage. Cu(Ⅱ) complexes disrupt redox homeostasis by GSH depletion and •OH generation, downregulate GPX4 and SLC7A11 expression, and induce ferroptosis with DAMP release (Huang K. B. et al., 2024). Self-assembled copper-alanine NPs (CACG) deplete GSH through reduction from Cu^2+^ to Cu^+^. The resulting Cu^+^ catalyzes H_2_O_2_ conversion to ROS, downregulates GPX4, and upregulates ACSL4 to trigger ferroptosis. Dying cells upregulate CHOP to induce ER stress and CRT exposure, ultimately activating anti-tumor immunity (Song et al., 2023). Cu^2+^/Cu^+^ in HCuS-PE@TSL-tlyp-1 generates •OH via Fenton reactions, accumulating lipid peroxide to induce ferroptosis and DAMP release (Wang et al., 2024b). FG-CDs@Cu depletes GSH via Cu^2+^, suppresses GPX4 expression, and generates lipid peroxide to drive ferroptosis and CRT translocation (Bao et al., 2023a). Cu_2-x_S-GOx@CaCO_3_ produces cytotoxic •OH through Cu_2-x_S interaction with H_2_O_2_, synergizing with Ca^2+^ overload to cause mitochondrial dysfunction. This inhibits the GSH/GPX4 axis, accumulating lipid peroxide to induce ferroptosis and DAMP release (Huang et al., 2023). Bimetallic drug-gene co-delivery systems Cu/ZIF-8@U-104@siNFS1-HA and CISAR nanoplatform utilize Cu^2+^/Cu^+^ and Fe^3+^/Fe^2+^ to inhibit the GSH/GPX4 axis, activating immunogenic ferroptosis (Du et al., 2022; Wang Z. et al., 2024). Similarly, CuSe/CoSe_2_@syrosingopine (CSC@Syro) employs Cu^+^/Cu^2+^ and Co^2+^/Co^3+^ to suppress GSH/GPX4 and trigger immunogenic ferroptosis (Yang et al., 2023).

### 2.4 Induction of tumor cell cuproptosis

 In 2022, Tsvetkov et al. discovered that copper binds to lipoylated proteins in the tricarboxylic acid cycle, inducing their aggregation and iron-sulfur cluster loss, which triggers proteotoxic stress and ultimately leads to cuproptosis (Tsvetkov et al., 2022). Key events driving cuproptosis include dysregulated copper transporters, enhanced ionophore-mediated transport, and GSH depletion. These processes collectively elevate intracellular Cu^2+^/Cu^+^ levels, establishing a mechanistic framework for copper-mediated regulation of cell fate. Cuproptosis-related genes comprise two categories: the lipoic acid pathway and the pyruvate dehydrogenase complex (Xie J. et al., 2023). Notably, while oxidative stress from Fenton-like activity of copper is not the initial event of cuproptosis, it actually accelerates the process. Oxidative stress sensitizes cuproptosis by disrupting antioxidant defenses (e.g., GSH depletion), causing mitochondrial dysfunction that impairs ATP-dependent copper transport, and directly damaging copper transporters (Liu et al., 2024a; Vo et al., 2024). Crucially, copper-based nanomedicine-modulated oxidative stress enhances tumor immunogenicity through DAMP release and immune cell stimulation.

MACuS promotes ROS generation via a dynamic reaction between Cu^2+^/Cu^+^ and GSH to intensify cuproptosis and release DAMPs, reprogramming the immunosuppressive TIME (Zu et al., 2024). CEL NPs release Cu^2+^ and ES under NIR-Ⅱ irradiation, inducing cuproptosis through ferredoxin 1 (FDX1) and lipoic acid synthetase (LIAS) downregulation amplified by GSH depletion; toxic aggregation of dihydrollipoamide S-acetyltransferase (DLAT) increases DAMP release to activate anti-tumor immunity (Cheng et al., 2025). Zn-Cu bimetallic nanomedicine CZP NPs provide sustained Cu^+^ to catalyze •OH generation, causing mitochondrial damage and downregulating Fe-S cluster proteins, ultimately releasing mitochondrial DNA (mtDNA) to activate the cGAS-STING pathway and enhance immunotherapy in triple-negative breast cancer. Activation of the cGAS-STING pathway enhances the maturation and activation of antigen-presenting cells by promoting the secretion of cytokines such as type I interferons. STING activation not only stimulates type I interferon secretion through the TBK1/IRF3 axis but also triggers NF-κB activation to regulate PD-L1 expression on tumor cells. The average PD-L1 expression on 4T1 cells treated with CZP NPs was significantly higher than that in the control group (Zhou B. et al., 2025). Similarly, CGNPs utilize Cu^2+^/Cu^+^ to generate ROS, thereby amplifying cuproptosis in triple-negative breast cancer. Mitochondrial disruption leads to the release of mtDNA, which activates the cGAS–STING pathway and promotes the release of DAMPs. The release levels of IFN-β (a representative type I interferon cytokine) and IL-6 were both elevated in the 4T1 cell group treated with CGNPs(Xu et al., 2025). Cu-MOF and Cu-IR783 NPs downregulate LIAS and FDX1 expression while promoting DLAT aggregation to induce cuproptosis. Cu^+^-mediated ROS generation and DAMP release remodel the TIME, enabling primary and metastatic tumor treatment (Hu J. et al., 2024; Cao et al., 2025). Cu/TI mediates cuproptosis via mitochondrial oxidative stress, DLAT aggregation, and FDX1 downregulation, releasing DAMPs to reverse the low immunogenicity of cancer cells (Li et al., 2024c). ECNM disrupts redox homeostasis via GSH depletion and ROS generation, synergizing with FDX1/DLAT suppression-induced cuproptosis to release DAMPs and activate antitumor immunity (Wu et al., 2025). Cu_2-x_Se@cMOF releases Cu^+^ to generate ROS and deplete GSH, enhancing cuproptosis and DAMP release, achieving synergistic immunotherapy with anti-PD-L1 (Zhao C. et al., 2024). PCD@CM releases Cu^2+^, which reacts with the excess GSH in the TME, leading to GSH depletion. Meanwhile, Cu^+^-mediated ROS generation amplifies cuproptosis. These mechanisms synergistically promote the release of DAMPs, thereby enhancing the efficacy of anti-PD-L1 therapy (Dai et al., 2024).

ES-Cu-MOF nanomedicine releases Cu^2+^ and ES in fibrosarcoma cells; Elesclomol (ES) shuttles Cu^2+^ to mitochondria, increasing mtROS, reducing DLAT/FDX1 expression, and inducing immunogenic cuproptosis with DAMP release (Luo et al., 2024). PEG@Cu_2_O-ES generates abundant ROS to damage ATP-dependent copper transporters (ATP7A/B) on cancer cell membranes, reducing copper efflux to amplify cuproptosis and remodel the TIME for enhanced immunity (Li W. et al., 2024). CLDCu releases DSF and Cu^2+^ to form bis(diethyldithiocarbamate)-copper (CuET), generating excessive ROS that causes mitochondrial damage and ATP depletion to inhibit ATP7B, enhancing Lip-DLAT/DLAT/FDX1 downregulation-mediated cuproptosis with DAMP release (Yan et al., 2024). Similarly, PDA-DTC/Cu NPs release excess Cu^2+^ to produce ROS that disrupts mitochondrial function and limits ATP supply, thereby inhibiting copper transporters ATP7A/B. This process further enhances cuproptosis via DLAT aggregation and Fe-S cluster destabilization, while releasing DAMPs to promote DC maturation and activate infiltrating T cells (Chang et al., 2024). Mito-Jammer decomposes in the acidic TME to release CaO_2_ and Cu^2+^; CaO_2_-generated H_2_O_2_ and Ca^2+^ overload intensify Cu^2+^-derived ROS, creating a storm that damages mitochondria and depletes ATP to block Cu-ATPase activity, amplifying cuproptosis and DAMP release (Du C. et al., 2024).

As a newly identified form of RCD, the interplay between cuproptosis and copper-based nanomedicine-modulated oxidative stress warrants further exploration. These innovative strategies for activating anti-tumor immunity offer new insights into the role of copper-dependent redox regulation within TIME.

### 2.5 Induction of multiple RCD modalities in tumor cell

Extensive molecular crosstalk exists among various RCD forms, with shared upstream signaling pathways or common regulatory molecules determining cell fate through critical molecular switches (He et al., 2024). Within the complex landscape of tumor immunity, multiple RCD modalities may coexist due to the spatiotemporal heterogeneity of the TIME and specific therapeutic interventions (He et al., 2024). This intricate interplay reflects traditional research approaches that categorize cell death into distinct modalities—a simplification that potentially overlooks the reality of cellular states. Beyond established RCD pathways, copper-based nanomedicine-induced oxidative stress critically bridges multiple RCD forms and tumor immunogenic activation.

PANoptosis, an inflammatory RCD pathway involving caspase and RIPK activation, is regulated by the PANoptosome. Zn–CuO_2_ NPs disrupt intracellular Cu^2+^/Zn^2+^ homeostasis, leading to abnormal accumulation of metal ions. Cu^2+^ synergizes with Zn^2+^ overload to exacerbate mitochondrial dysfunction through Fenton-like reactions, thereby enhancing ROS accumulation, which accelerates mitochondrial protein/mtDNA release, upregulates Bax, downregulates Bcl-2, and triggers cytochrome c release. Excess Cu^2+^/Zn^2+^ induces mitochondrial damage, dsDNA release, and ER stress, activating AIM2-PANoptosome-mediated PANoptosis and releasing DAMPs to remodel TIME (Hou et al., 2024). Based on the aforementioned findings, copper-based nanomedicine-induced oxidative stress plays a pivotal role in linking multiple RCD modalities with tumor immunogenicity. Notably, such oxidative stress may, in certain contexts, simultaneously activate two or more RCD pathways, thereby enhancing immunogenic responses. With researchers’ talents and efforts, recent advances have demonstrated that the precise coordination of crosstalk among these RCD mechanisms offers new paradigms for restoring tumor immunogenicity.

Given copper’s centrality to cuproptosis, integrating copper-based nanomedicine-induced oxidative stress with other RCD forms has emerged as a main strategy. These nanomedicines disrupt redox homeostasis to induce apoptosis, pyroptosis, or ferroptosis while enabling cuproptosis-centered combinations. PEG-DTPA-SS-CPT releases Cu^2+^, depletes GSH to generate toxic Cu^+^ and ROS, and synergizes with doxorubicin (DOX)/camptothecin to upregulate Bax/cleaved caspase-3 while downregulating Bcl-XL, inducing apoptosis in breast cancer. Copper-driven oxidative stress and GSH depletion further enhance cuproptosis via copper accumulation, releasing DAMPs for antitumor immunity (Wang N. et al., 2024). BCO-V_Cu_ triggers ROS bursts to upregulate Bax and downregulate Bcl-2, inducing apoptosis, while its ferroelectric properties enhance Cu^2+^ release/aggregation through membrane permeability alterations; Cu^2+^ accumulation downregulates FDX1/LIAS, promoting DLAT oligomerization and cuproptosis, ultimately enhancing DAMP release and immune activation (Du Y. et al., 2024). FA-PZ@MOF NPs promote the accumulation of Zn^2+^ and Cu^+^, exacerbating ROS storms and mitochondrial damage. This leads to lipoylated protein aggregation, Fe–S cluster loss, and the induction of cuproptosis. Meanwhile, ROS and Polyphyllin Ⅵ act synergistically to activate the NLRP3 inflammasome, recruit caspase-1, and cleave GSDMD, thereby triggering pyroptosis. The enhanced interplay between cuproptosis and pyroptosis promotes the release of DAMPs and anti-tumor immunity (Yi et al., 2025). F127 MOF-199 induces cuproptosis via copper overload, while *in situ* sulfidation converts it to Cu_2-x_S NPs, activating photothermal therapy (PTT)/chemodynamic therapy (CDT) to release cytochrome c, activate caspase-3, and cleave GSDME for pyroptosis, culminating in DAMP release (Xiao W. et al., 2024).

The combination of ferroptosis and cuproptosis strategies further highlights the pivotal role of copper-based nanomedicines in tumor immune regulation. Cu-DBCO/CL nanozymes generate ROS storms through POD-like activity, leading to GSH/GPX4 depletion, lipid peroxidation, and ferroptosis. Simultaneously, mitochondrial dysfunction reduces ATP levels, impairing ATP7A-mediated copper efflux and resulting in intracellular Cu^+^ accumulation. The trapped Cu^+^ promotes DLAT oligomerization and downregulation of LIAS, thereby promoting cancer cell death and facilitating the release of DAMPs and TAAs (Liu et al., 2024c). Moreover, oxidative stress modulated by copper-based nanomedicines may induce the interplay among three distinct forms of RCD within tumor cells. NSeMON-P@CuT/LipD releases copper, which induces DLAT aggregation and Fe–S cluster loss, thereby triggering cuproptosis. Concurrently, Cu^2+^/Cu^+^ depletes GSH and generates •OH, inducing ferroptosis through the GSH/GPX4 pathway. The resulting oxidative stress further amplifies cuproptosis by exacerbating mitochondrial dysfunction and inhibiting ATP7B-mediated copper efflux. In addition, pemetrexed acts synergistically with •OH to induce apoptosis (Zhang M. et al., 2025). These coordinated RCD cascades release DAMPs to activate antitumor immunity (Luo et al., 2025).

Despite much progress, the detailed molecular mechanisms by which copper-based nanomedicines modulate oxidative stress across various RCD modalities remain incompletely understood, particularly with regard to copper’s unique biological roles. The potential impact of copper-based nanomedicine-modulated oxidative stress on other forms of RCD, such as necroptosis and secondary necrosis, calls for further exploration. Progress in these areas will provide deep insights into copper-driven ICD and support the development of advanced cancer immunotherapies (Liu et al., 2024b).

## 3 Co-delivery strategies for tumor immunotherapy

Effective drug delivery is essential for precision and potent immunotherapy. Elevating intracellular Cu^2+^ levels represents a promising strategy to advance copper-based nanomedicine-modulated oxidative stress and enhance tumor immunotherapy. Furthermore, leveraging nanomedicine delivery platforms enables robust immunotherapeutic enhancement through co-delivery strategies, which improve the efficacy of copper-based nanomedicines while reducing adverse effects (Liu C. et al., 2023; Wadhwa et al., 2024). Due to the characteristic features of the TME, which result from highly permeable vascular systems, dense extracellular matrix, stromal cells, and dysfunctional lymphatic networks, the size effect of nanomedicines plays a crucial role in tumor targeting. Size serves as a key parameter governing nanomedicines, as it determines the surface-to-volume ratio, thereby influencing the density of active sites per unit volume and ultimately dictating their catalytic activity. Generally, nanomedicines within the size range of 1–100 nm possess a high surface-area-to-volume ratio, which are densely packed with active sites that enhance catalytic efficiency and promote ROS generation (Wang Y. et al., 2024). The small size of nanoparticles facilitates improved tissue permeability and drug delivery. However, smaller nanomedicines typically exhibit a shortened half-life in the bloodstream and rapid elimination from the body, leading to reduced tumor accumulation. The unique size-dependent targeting efficiency and catalytic efficiency form the core of drug co-delivery strategies (Xu et al., 2023). Moreover, such delivery strategies can be further optimized through surface modifications. Benefiting from surface modification, copper-based nanomedicines achieve deep intratumoral penetration while preserving their intrinsic properties. These modification strategies are typically categorized into non-biomimetic, partially biomimetic, and fully biomimetic approaches. Non-biomimetic strategies alter physicochemical surface properties such as charge, shape, hydrophobicity, and softness. Partially biomimetic approaches leverage overexpressed receptors, essential nutrient mimicry (e.g., albumin), tumor-homing peptides, and tumor-penetrating peptides to enhance deep tumor targeting. Fully biomimetic strategies utilize permeable cells or cell-derived entities for tumor penetration, with biomimetic systems like tumor cell membranes and extracellular vesicles generally offering advantages in safety and simplicity (Li et al., 2020). Although the penetration-enhancing effects across different modification strategies cannot be directly compared, surface engineering enables copper-based nanomedicines to achieve high solubility, superior stability, and precise drug delivery, thereby enhancing therapeutic efficacy while minimizing systemic toxicity.

### 3.1 Copper ionophores

Copper ionophores, such as ES and DSF, have been applied for Cu^2+^ delivery in tumor immunotherapy. ES stands out for its exceptional transport rate and selective mitochondrial copper delivery. Nanomedicine encapsulation of ES-Cu complexes enhances circulatory stability and tumor targeting of ES. For instance, ECNM employs a ROS-responsive polymer to encapsulate ES-Cu, prolonging systemic circulation and boosting tumor accumulation. Subsequently, released ES and Cu^2+^ synergize with cinnamaldehyde to disrupt redox homeostasis, inducing cuproptosis and DAMP release, thereby achieving immunotherapy (Table 1) (Wu et al., 2025). Similarly, TSF@ES-Cu NPs generate abundant ROS, disrupt copper homeostasis, induce cuproptosis, and facilitate the release of DAMPs. These effects collectively promote DCs maturation, enhance CD8^+^ T-cell infiltration, and reduce M2 tumor-associated macrophages (TAMs), thereby enhancing anti-tumor immune responses (Table 1) (Gao et al., 2025). PEG@Cu_2_O-ES passively targets breast cancer via the enhanced permeability and retention (EPR) effect, releasing ES and Cu_2_O. ROS derived from Cu_2_O attack ATP-dependent copper transporters on cancer cell membranes, reducing Cu^2+^ efflux, while free ES boosts Cu^2+^ influx, synergistically amplifying cuproptosis. Additionally, Cu^2+^ upregulates PD-L1 expression, sensitizing tumors to anti-programmed cell death protein 1 (PD-1) immunotherapy (Table 1) (Li W. et al., 2024).

**TABLE 1:** Copper-based nanomedicine Co-delivery strategies for enhanced tumor immunotherapy.

Formulations	Drug	Cu-based nanomedicine	Surface modification	Mechanism	Cell type	Application	Ref.
ECNM	ES NLG919	ES-Cu	4T1 membrane	Cuproptosis TIM ↓	4T1	Breast cancer	
TSF@ES-Cu NPs	ES	ES-Cu	Tussah silk fibroin contain arginine-glycine-aspartic acid tripeptides	Cuproptosis	Mia,Panc-1,Pan02	Pancreatic Cancer	Gao et al. (2025)
PEG@Cu_2_O-ES	ES	Cu_2_O	PEG	Cuproptosis	4T1	Breast cancer anti-PD-L1 therapy	Li et al. (2024a)
CuCH-NCs	DSF	Copper carbonate hydroxide nanocrystals	Albumin	Apoptosis	4T1	Breast cancer anti-PD-L1 therapy	Li et al. (2023)
Alb/LF NPs	DDC	DDC/Cu-Fe	Albumin, lactoferrin	Ferroptosis	GL261	Glioma	Wang et al. (2024d)
PDA-DTC/Cu NPs	DTC	Cu(DTC)_2_	None	Cuproptosis	4T1	Breast cancer	Chang et al. (2024)
PCD@CM	DOX	Cu-Pdots	4T1 membrane	Cuproptosis	4T1	Breast cancer anti-PD-L1 therapy	Dai et al. (2024)
M/A@MOF@CM	MTO Axitinib	Cu-based MOF nanoparticle	4T1 membrane	Cuproptosis Ferroptosis Apoptosis	4T1	Breast cancer	Ji et al. (2024)
PdPtCu/NLG919@BSA-Ce6/T^ER^	NLG919	PdPtCu nanozyme	Bovine serum albumin	Tryptophan/kynurenine↑	4T1	Breast cancer	Xie et al. (2023b)
CSC@Syro	Syrosingopine	CuSe	Bovine serum albumin	Ferroptosis Lactate↓	4T1	Breast cancer	Yang et al. (2023)
Cu-DBCO/CL	CHO LOX-IN-3	Cu-DBCO	None	Cuproptosis Ferroptosis ECM remodeling PD-1↓ TIM-3↓	4T1	Breast cancer	Liu et al. (2024c)
PCB	BPTES	CuP	Platelet membrane	Cuproptosis	4T1	Breast cancer	Zhang et al. (2024)
LipoCu-OA/ACF	Acriflavine	Cu-OA	Liposome	HIF-1↓ Lactate↓ Adenosine↓ PD-L1↓	4T1	Breast cancer	Zhang et al. (2023a)
Cu-Pic/HA NPs	Piceatannol	Cu-Pic	Hyaluronic acid	Pyroptosis Cuproptosis	4T1 MCF-7	Breast cancer	Zhu et al. (2024)
CMO-R@4T1	R848	Cu-MoOx	4T1 membrane	TLR7/8 activation	4T1	Breast cancer	Jana et al. (2024)
GCT@CMNPs	Toyocamycin	1G_3_-Cu	B16 membrane	Apoptosis ER stress↑	B16	Melanoma anti-PD-L1 therapy	Guo et al. (2022)
S@Cu-MOF/PPI	Polyphyllin I	Cu-MOF	None	Apoptosis Cuproptosis cGAS/STING↑	4T1	Breast cancer anti-PD-L1 therapy	Xu et al. (2024)

DSF, an FDA-approved anti-alcoholism agent, exerts antitumor effects primarily through ROS modulation, with its efficacy dependent on copper. *In vivo*, DSF metabolizes into diethyldithiocarbamate (DTC), which chelates Cu^2+^ to form the cytotoxic complex Cu(DTC)_2_. Co-delivering DSF, DDC, or DSF prodrugs with copper markedly enhances bioavailability and therapeutic potency of copper-based nanomedicines (Chen et al., 2022; Chang et al., 2024). CuCH-NCs/DSF releases Cu^2+^ to modulate oxidative stress, which subsequently complexes *in situ* with DSF to form the highly cytotoxic compound Cu(DTC)_2_. This process induces robust cuproptosis, upregulates PD-L1 expression, and promotes the release of DAMPs, thereby synergizing with anti-PD-L1 immunotherapy to enhance anti-tumor immunity (Table 1) (Li et al., 2023). Alb/LF NPs encapsulating DDC/Cu-Fe facilitate brain accumulation, where DDC/Cu-Fe cooperatively induces glioma cell ferroptosis through oxidative stress, activates T cell immunity, and suppresses TAMs by FROUNT (also known as NUP85) inhibition (Table 1) (Wang R. et al., 2024). PDA-DTC/Cu NPs efficiently deliver Cu^2+^ for intracellular accumulation by employing polydopamine-coated nanocarriers, releasing DTC and Cu^2+^ within tumors via the EPR effect. These NPs promote cuproptosis through ROS generation and copper overload, leading to DAMP release that promotes DC maturation and T-cell activation. Meanwhile, generated ROS reprograms TAMs toward anti-tumor phenotype (Table 1) (Chang et al., 2024).

### 3.2 Chemotherapeutic drug

Anthracycline chemotherapeutics are extensively proven to alleviate tumor-induced immunosuppression and activate antitumor immunity. Copper-based nanomedicine co-delivery strategies with anthracycline chemotherapeutics not only reduce off-target toxicity but also enhance tumor cell eradication through immunogenic activation. DOX initiates mitochondrial damage by binding to cardiolipin on inner mitochondrial membranes, generating ROS that synergize with oxidative stress modulated by copper-based nanomedicine for immunotherapy. In PCD@CM, Cu^2+^ and DOX are co-delivered via a homologous targeting strategy enabled by tumor cell membrane encapsulation. Upon GSH-mediated reduction of Cu^2+^ to Cu^+^, PCD@CM undergoes responsive disassembly, releasing Pdots and DOX. The released Cu^+^ not only catalyzes the Fenton-like reaction with endogenous H_2_O_2_ to generate •OH, inducing intracellular oxidative stress, but also triggers cuproptosis. Furthermore, the synergistic effect of NIR-Ⅱ PTT, chemotherapy, and cuproptosis promotes the release of DAMPs and TAAs, thereby initiating a potent antitumor immune response through DC maturation (Table 1) (Dai et al., 2024). In particular, the anthracycline derivative mitoxantrone (MTO) exhibits exceptional photothermal conversion capabilities. M/A@MOF@CM NPs, comprising copper-based metal-organic frameworks (MOF) loaded with MTO and axitinib, induce ferroptosis through copper-modulated oxidative stress. This system disrupts copper homeostasis by impairing ATP production, thereby increasing cytoplasmic Cu^2+^ levels and initiating cuproptosis. Simultaneously, the released MTO exerts both chemotherapeutic and PTT effects. M/A@MOF@CM effectively promotes cancer cell death and DAMP release, contributing to the reprogramming of the immunosuppressive TIME (Table 1) (Ji et al., 2024).

### 3.3 Metabolic intervention drug

Tumor cells exhibit hyperactive metabolism compared to normal cells, leading to metabolic waste accumulation and acidic pH in the TME. This metabolic heterogeneity not only supports tumor proliferation but also facilitates immune evasion. Combining copper-based nanomedicines with metabolic intervention drugs effectively reverses immunosuppressive TIME.

Indoleamine 2,3-dioxygenase 1 (IDO 1)-driven tryptophan catabolism via the kynurenine pathway creates an immunosuppressive environment that facilitates immune evasion and promotes tumor progression. In PdPtCu/NLG919@BSA-Ce6/T^ER^, the IDO inhibitor NLG919 blocks the tryptophan/kynurenine immune escape axis, enhancing effector T-cell function while suppressing regulatory T-cells, thereby amplifying tumor-specific immunity induced by PdPtCu-modulated oxidative stress (Table 1) (Xie Y. et al., 2023). CuSe/CoSe_2_@syrosingopine downregulates monocarboxylate transporter 4 (MCT4) to modulate lactate metabolism in cancer cells. This acidifies the intracellular milieu, enhancing Cu^+^/Cu^2+^ and Co^2+^/Co^3+^ catalytic ROS generation to induce ferroptosis, while neutralizing acidic TME to alleviate immunosuppression (Table 1) (Yang et al., 2023). Cu-DBCO/CL co-delivers cholesterol oxidase (CHO) and the lysyl oxidase inhibitor LOX-IN-3. Benefiting from its enzyme-like activity, Cu-DBCO/CL catalyzes O_2_ and H_2_O_2_ into O_2_^−^• and •OH, promoting both cuproptosis and ferroptosis. ROS-triggered release of LOX-IN-3 inhibits lysyl oxidase activity, remodeling the extracellular matrix, and enhancing CD8^+^ T cell infiltration. Furthermore, CHO-induced cholesterol depletion not only amplifies •OH generation but also downregulates immune checkpoints PD-1 and TIM-3, thereby restoring CD8^+^ T-cell antitumor activity (Table 1) (Liu et al., 2024c). BPTES, a selective glutaminase (GLS1) inhibitor, suppresses GSH synthesis. Platelet membrane-coated PCB delivers BPTES to tumors, where it inhibits GLS expression and depletes GSH, promoting cuproptosis and copper-mediated ROS production. This dual mechanism triggers tumor cell death and DAMP release, increasing mature DCs and CD8^+^ T cell infiltration (Table 1) (Zhang et al., 2024). LipoCu-OA/ACF, co-assembled from copper oleate and the HIF-1α inhibitor acriflavine (ACF), releases Cu^2+^ to activate immune responses through oxidative stress. Simultaneously, ACF depletes GSH, inhibiting HIF-1α signaling and amplifying oxidative stress. Beyond HIF-1α suppression, ACF alleviates immunosuppression by reducing extracellular lactate and adenosine levels, as well as downregulating PD-L1 expression (Table 1) (Zhang X. et al., 2023). Pic, an arginase 2 (Arg2) inhibitor, targets mitochondrial Arg2 to disrupt polyamine synthesis. Cu-Pic/HA NPs generate ROS via multiple enzyme-like activities while depleting intracellular polyamines. Released Cu^2+^ induces lysosomal disruption, inactivating the polyamine transporter ATP13A2 on lysosomal membranes and blocking polyamine uptake. Meanwhile, Pic inhibits Arg2 activity, halting upstream polyamine synthesis and promoting mitochondrial ROS accumulation. The effective synergy between pyroptosis and cuproptosis triggers DAMP release, activating anti-tumor immunity (Table 1) (Zhu et al., 2024).

### 3.4 Immunostimulant

To enhance the efficacy of oxidative stress modulated by copper-based nanomedicines in activating tumor immunity, researchers co-deliver nanomedicines with immunotherapeutic agents such as Toll-like receptor (TLR) agonists. The TLR7/8 agonist resiquimod (R848) primarily targets antigen-presenting cells like DCs, promoting the secretion of pro-inflammatory cytokines and improving antigen-presenting cell polarization/maturation to initiate T-cell responses. Although TLR agonists contribute to remodeling the TIME, their toxicity due to excessive cytokine production remains a concern. Nanoplatforms integrating TLR agonists with copper-based nanomedicines effectively reduce off-target toxicity while amplifying oxidative stress-modulated immunogenic activation.

CMO-R@4T1 utilizes Cu-MoO_x_ nanozymes to generate ROS and disrupt antioxidant defenses, causing oxidative damage to tumor cells and releasing DAMPs and TAAs. Released R848 triggers immune activation, synergistically eliminating tumors and establishing long-term immune memory (Table 1) (Jana et al., 2024). Toyocamycin amplifies ER stress by targeting the IRE1α–X–box binding protein 1 (XBP1) pathway. In GCT NPs co-loaded with 1G_3_-Cu and toyocamycin, 1G_3_-Cu-induced mitochondrial dysfunction synergizes with toyocamycin-mediated ER stress to drive tumor cell apoptosis and DAMPs release, effectively inhibiting tumor growth, promoting DC maturation, and increasing CTL infiltration (Table 1) (Guo et al., 2022). Polyphyllin I reverses immunosuppression by activating the cGAS–STING pathway. S@Cu-MOF/PPI delivers Polyphyllin I with reduced toxicity; released Cu^2+^ triggers mitochondrial dysfunction and cuproptosis. ROS generation and mtDNA release synergize with Polyphyllin I to activate the cGAS/STING pathway, enhancing anti-tumor immunity (Table 1) (Xu et al., 2024). Additionally, co-delivery of other immunostimulatory metal ions (e.g., Zn^2+^, Mn^2+^) with copper-based nanomedicines can further enhance innate immune responses by activating the cGAS–STING signaling pathway (Li Q. et al., 2025; Liu et al., 2025; Zhou B. et al., 2025).

## 4 Amplified oxidative stress for tumor immunotherapy

The core mechanism by which copper-based nanomedicines modulate oxidative stress in tumor immunotherapy is CDT, which exploits the unique redox metabolism of tumor cells to convert endogenous H_2_O_2_ into lethal •OH via Fenton-like reactions or enzyme-like activity. However, the efficacy of copper-induced oxidative stress in tumor immunotherapy is limited by the intrinsic TME, including its weak acidity (pH 6.5–6.8), insufficient H_2_O_2_ supply (50–100 μM), and robust antioxidant defenses such as GSH. The valence state of copper determines the catalytic ability of copper-based nanomedicines to generate ROS. The Fenton reaction kinetics of Cu^+^ is 1 × 10^4^ M^-1^ s^-1^, higher than that of Cu^2+^ at 4.6 × 10^2^ M^-1^ s^-1^ (Hao et al., 2021). Experimental results indicate that Cu-doped hollow carbon spheres (Cu^0^) exhibit stronger POD activity than CuO-adhered hollow carbon spheres (Cu^2+^) (Xu et al., 2023). Theoretical calculations reveal that Cu^0^ and Cu^+^ enhance substrate adsorption capacity, while Cu^2+^ contributes to lowering the activation energy barrier (Mao et al., 2025).

Furthermore, the diverse forms of copper-based nanomedicines, including complexes, hydroxides, oxides, sulfides, and MOFs, endow these nanomaterials with excellent responsiveness to stimuli like light, ultrasound, and magnetic fields. Copper oxides possess characteristics such as ease of storage, high stability, good tunability, controllable catalytic sites, surface functionalizability, and low cost. The formation of oxygen vacancies in copper oxides facilitates accelerated electron transfer rates and enhanced substrate adsorption, thereby improving catalytic activity (Liu Q. et al., 2021). Copper chalcogenides are p-type semiconductors that exhibit strong localized surface plasmon resonance due to copper deficiencies. The abundant free carriers in Cu_2–xE_ (E = S, Se, Te, 0 ≤ x ≤ 1) significantly interact with the oscillating electromagnetic field of incident light. Cu_2–xE_ species demonstrate high near-infrared light absorption across the relatively broad wavelength ranges of the NIR-I (750–900 nm) and NIR-II (1,000–1,700 nm) biological windows, enabling copper chalcogenides to enhance ROS generation capability through photo-stimulation (Zhao et al., 2020). MOFs, as a relatively new class of porous solid materials, have garnered extensive research interest in catalysis due to their well-defined coordination networks, mesoporous structures, and tunable porosity. MOFs are crystalline materials composed of metal ions or clusters connected by organic ligands. Copper can serve as the catalytically active center in MOFs to form copper-based MOF nanomedicines, whose porous structures enable rapid mass transfer and achieve optimal adsorption and separation of target molecules through adjustable pore sizes (Li et al., 2024b). Among these, copper single-atom nanomedicines represent the forefront of biocatalysis. The isolated dispersion of active catalytic sites ensures maximum utilization efficiency of metal atoms. Additionally, their tunable coordination environments and electronic structures facilitate a deeper understanding of catalytic mechanisms (Peng et al., 2024). Additionally, some copper-based nanoplatforms function as effective auxiliary agent carriers. These properties enable oxidative stress modulated by copper-based nanomedicines to be synergistically combined with other therapies as “1 + X” strategies for tumor immunotherapy, demonstrating superior immune activation compared to monotherapies.

### 4.1 Enhanced fenton-like reactions

The insufficient intracellular H_2_O_2_ levels and weakly acidic environment within tumor cells are major limiting factors for ROS generation by copper-based nanomedicines, resulting in immune activation that is insufficient for therapeutic efficacy. Within this complex TIME, increasing the concentration of the catalytic substrate H_2_O_2_ and enhancing acidity are the primary strategies for improving the overall efficiency of Fenton-like reactions. Specifically, elevating H_2_O_2_ concentration can be achieved through increasing its supply and reducing its consumption, while strengthening the acidic environment can be realized by boosting the levels of acidic substances within the TIME. Approaches to increase H_2_O_2_ supply include exogenous H_2_O_2_ delivery and *in situ* catalytic generation via enzymatic reactions. Strategies to reduce H_2_O_2_ consumption involve depleting GSH to minimize H_2_O_2_ loss and inhibiting its decomposition.

#### 4.1.1 Supply of H_2_O_2_ through exogenous substances

Peroxides composed of metal ions and peroxide groups, such as CaO_2_, CuO_2_, and certain bimetallic peroxides, release H_2_O_2_ directly within the TME without external stimulation, serving as substrates for Fenton-like reactions catalyzed by copper-based nanomedicines. In (Cu_2_Se-CaO_2_)@LA, CaO_2_ reacts with water to generate H_2_O_2_ and Ca^2+^; subsequently, Cu_2_Se converts H_2_O_2_ into •OH. The amplified ROS generation synergizes with Ca^2+^ overload to induce sequential damage to mitochondria and the ER. This triggers ICD via the ROS-PERK-eIF2α pathway, ultimately promoting DC maturation and CTL infiltration (Feng X. et al., 2023). Similarly, CaO_2_-CuO_2_@HA NC rapidly releases substantial amounts of Ca^2+^, Cu^2+^, and H_2_O_2_ within tumor cells. The Cu^2+^ consumes GSH and catalyzes H_2_O_2_ to generate •OH, synergizing with Ca^2+^ overload to cause mitochondrial damage (Liu B. et al., 2021). Ca^2+^ induced mitochondrial permeability transition pore opening with consequences of the release of cyt-c. Ca^2+^ overload enhances oxidative stress and mitochondrial damage within tumor cells, leading to downregulation of ATP levels and inhibition of Cu-ATPase activity, thereby potentiating cell death induced by copper-based nanomedicines. The combination of copper-based nanomedicines with CaO_2_ not only addresses the insufficient H_2_O_2_ supply but also synergizes with Ca^2+^ overload to sensitize tumor cells to death, releasing DAMPs and activating anti-tumor immunity (Du C. et al., 2024). Ce/Cu bimetallic peroxides decompose to generate H_2_O_2_, Cu^+^, and Ce^4+^, initiating a synergistic Ce^3+^/Cu^+^-mediated Fenton-like reaction that induces robust oxidative stress, thereby promoting the release of TAAs and DAMPs, which collectively enhance DC maturation, drive M1 polarization of TAMs, and activate effector T cells (Zhou H. et al., 2025). In Vk3@Si@CuZnIONP, vitamin K3 is reduced by NAD(P) H quinone oxidoreductase one within tumor cells to semiquinone and hydroquinone, which can subsequently be re-oxidized to quinone, generating substantial amounts of H_2_O_2_. Simultaneously, vitamin K3 supplementation increases intracellular H_2_O_2_ levels, while CuZnIONP catalyzes ROS production, inducing apoptosis and upregulating heat shock proteins and pro-inflammatory cytokines, thereby activating anti-tumor immunity (Chauhan et al., 2023).

#### 4.1.2 Self-supply of H_2_O_2_ through enzyme-catalytic systems

Inspired by cascade catalytic systems in living organisms, scientists have meticulously designed numerous cascade reaction platforms based on abundant tumor metabolites like glucose, pyruvate, and lactate. These systems are broadly categorized into natural enzyme-nanozyme and nanozyme-nanozyme catalytic systems. The natural enzyme glucose oxidase (GOx) oxidizes glucose into gluconic acid and H_2_O_2_, continuously supplying substrate and creating a more acidic environment to enhance Fenton-like reactions, thereby strengthening the ability of copper-based nanomedicines to activate tumor immunity via amplified oxidative stress.

GOx in CACG supplies H_2_O_2_ for Fenton-like reactions mediated by Cu^+^, generating substantial ROS. Increased intracellular oxidative stress induces ER stress and ferroptosis, promoting CRT exposure and further activating immunity (Song et al., 2023). GOx-loaded hCZAG effectively elevates intracellular H_2_O_2_ levels, consumes GSH via Cu^2+^, and promotes ROS generation through Cu^+^/Cu^2+^ cycling. Amplfied oxidative stress activates caspase-1, leading to GSDMD cleavage, inducing pyroptosis and DAMP release (Wang Y. Y. et al., 2024). Inspired by natural biomineralization in biological systems, scientists utilize calcium-based biomineralized nanomaterials to protect GOx from deactivation. Furthermore, GOx combines metabolic therapy with oxidative stress therapy by depleting glucose to disrupt tumor cell metabolism, impair the pentose phosphate pathway, inhibit the synthesis of reduced GSH and the CoQ_10_/CoQ_10_H_2_ redox cycle, simultaneously suppressing the GPX4/GSH and FSP1/CoQ_10_H_2_ antioxidant pathways. The amplified oxidative stress, coupled with compromised antioxidant defenses, induces ferroptosis and promotes the release of DAMPs, thereby recruiting CTLs (Huang et al., 2023). CQG NPs mimic GPx, CAT, SOD, and POD-like activities to deplete GSH, produce O_2_, and generate ROS, while the loaded GOx consumes glucose to mediate starvation therapy. These synergistic effects induce tumor cell pyroptosis and cuproptosis, releasing DAMPs to reprogram the immunosuppressive TIME and elicit potent anti-tumor immune responses *in vivo* (Qiao et al., 2023).

Au NPs, possessing GOx-like activity, enable some copper-based nanomedicines to achieve *in situ* H_2_O_2_ self-supply via a nanozyme-nanozyme system, inducing potent oxidative stress to activate immunity. In Cu_2_O@Au, Cu^2+^/Cu^+^ facilitates efficient Fenton-like reactions utilizing endogenous H_2_O_2_ and the lower acidity provided by the NP. Au NP-mediated glucose consumption disrupts the pentose phosphate pathway, reducing GSH synthesis, ultimately promoting DC maturation and T cell infiltration through ferroptosis (Lin et al., 2024).

Lactate oxidase (LOx) converts lactate in the TME into pyruvate and H_2_O_2_. This process not only enhances the oxidative stress generation capacity of copper-based nanomedicines but also alleviates the immunosuppressive microenvironment by depleting lactate. In PLNP Cu, Cu^+^ catalyzes H_2_O_2_ (supplied by LOx) to generate ⋅OH, inducing tumor cell death and releasing DAMPs to activate the immune system and inhibit tumors. Concurrently, intra- and extracellular lactate depletion remodels the immunosuppressive TME, inducing TAM polarization towards the M1 phenotype and activating immune cells (He et al., 2021). Similarly, in Cu-NS@UK@POx, pyruvate oxidase (POx) oxidizes excess intracellular pyruvate to generate large amounts of H_2_O_2_, thereby enhancing ROS-mediated pyroptosis and reprogramming the immune microenvironment. This includes M1 phenotype repolarization of macrophages and improved infiltration of CD4^+^ and CD8^+^ T cells, effectively treating tumors and activating systemic immune responses (Niu et al., 2024).

Regarding reducing H_2_O_2_ consumption, catalase inhibitors in CPTH can reduce H_2_O_2_ degradation, increasing mitochondrial H_2_O_2_ availability. The copper-based nanozymes in CPTH deplete mitochondrial GSH and exert peroxidase-like activity to induce ⋅OH generation, triggering mitochondrial oxidative damage and the release of TAAs and DAMPs (Lu et al., 2024). Additionally, inhibiting carbonic anhydrase IX (CA IX) can reverse intra- and extracellular pH gradients, promoting intracellular acidification and alleviating the extracellular acidic microenvironment. CuO_2_@G5-BS/TF not only lowers intracellular pH via a CA IX inhibitor but also achieves H_2_O_2_ self-supply through CuO_2_, ultimately enhancing the Fe^3+^/Cu^+^-mediated Fenton reaction (Huang H. et al., 2024).

### 4.2 Synergistic effects driven by external energy fields

**FIGURE 2:**
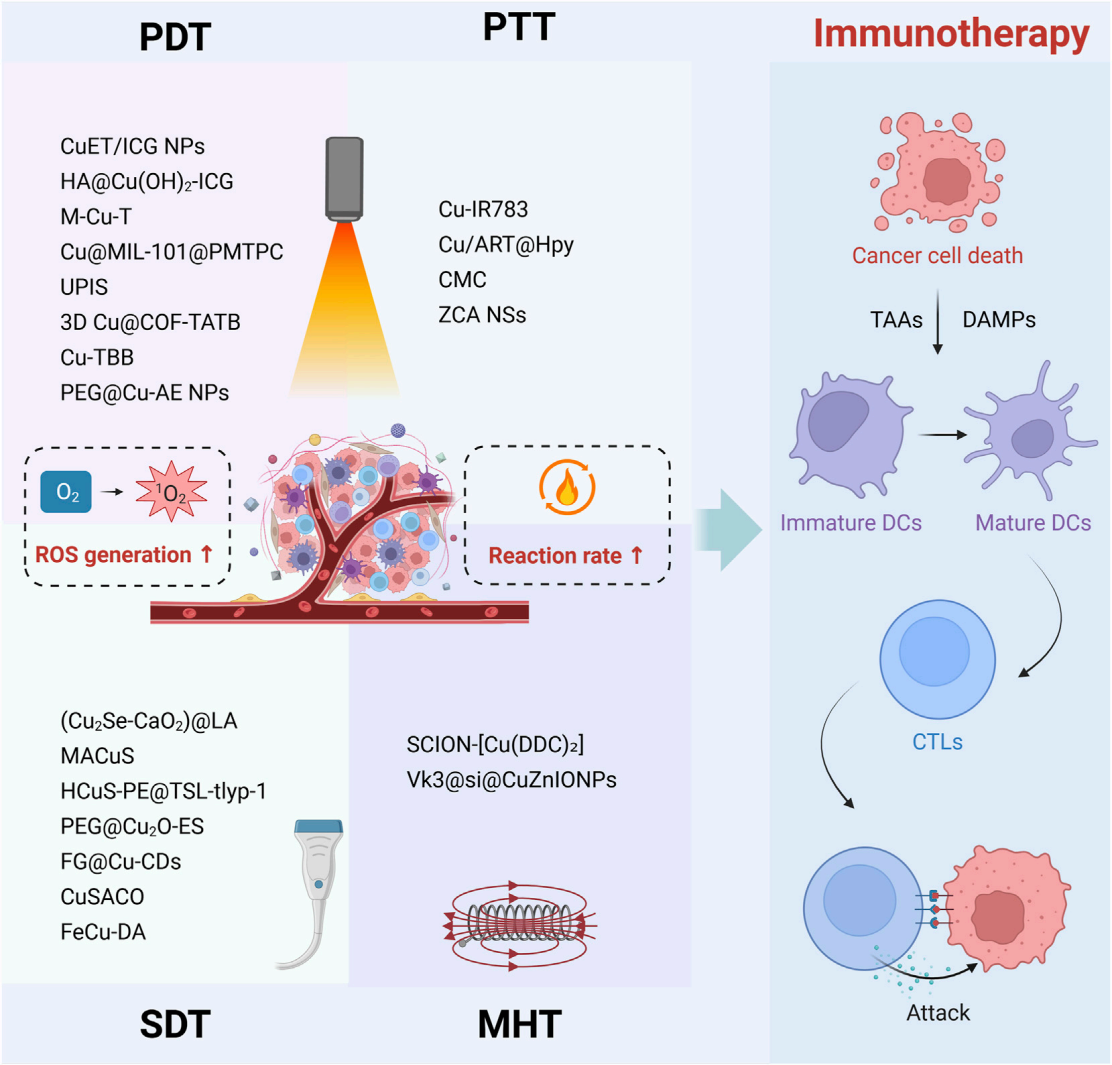
Multimodal immunotherapies of copper-based nanomedicine driven by external energy fields. Copper-based nanomedicine augments ROS generation through PDT and SDT, while simultaneously accelerating Fenton-like reaction rate via the heat produced by PTT and MHT. This ROS-amplified multimodal therapeutic strategy ultimately induces cancer cell death, releasing DAMPs and TAAs. These signals promote DC maturation and enhance CTL infiltration into tumor tissue, thereby enabling effective tumor cell clearance.

Currently, achieving satisfactory cancer immunotherapy using CDT based solely on Fenton-like reactions is challenging. External energy fields, including NIR light, ultrasound, magnetic fields, and microwaves, enhance the efficiency of copper-based nanomedicines in generating ROS for immune activation by directly converting external physical stimuli into chemical energy or indirectly augmenting Fenton-like reactions through heat generation (Figure 2). Compared to optimizing the endogenous reaction environment, utilizing external physical fields as remote or wireless energy sources allows for precise control over the progression and intensity of catalytic reactions, achieving a high degree of spatiotemporal controllability and tunable catalytic performance. Immunotherapy employing copper-based nanomedicines driven by external energy fields exhibits high selectivity, efficiency, and low side effects, making it a highly active research area in current tumor immunotherapy.

#### 4.2.1 Photosensitive copper-based nanomedicines

Photosensitizers utilize endogenous O_2_ within the TME to convert it into cytotoxic ^1^O_2_ under external laser irradiation for photodynamic therapy (PDT) (Xiao J. et al., 2024). NIR light offers high tissue penetration, and accordingly, various agents with NIR-responsive properties have been integrated as components of copper-based nanomedicines to regulate oxidative stress and enhance immunotherapy. Indocyanine dyes and porphyrins are two common types of photosensitizers combined with copper-based nanomedicines. Indocyanine dyes, including indocyanine green (ICG) and IR-820, assemble with copper-based nanomedicines to exert PDT effects for enhancing tumor immunity (Hou et al., 2022; Hu P. et al., 2024; Zhang Z. et al., 2025).

ICG, an FDA-approved clinical photosensitizer, addresses the poor solubility of CuET during delivery in CuET/ICG NPs. Here, ICG-generated ROS synergizes with CuET to induce tumor cell death, release DAMPs, and trigger mitochondrial dysfunction (Table 2) (Zhang Z. et al., 2025). In HA@Cu(OH)_2_-ICG, ICG is loaded into nHAC via sulfonic acid-Cu^2+^ coordination. The triple enzyme-like activities (POD, GSHOx, and CAT) of nHACI amplify oxidative stress, cooperating with ICG-mediated PDT to induce cell death and DAMP release. This downregulates M2 macrophage polarization and activates anti-tumor immunity (Table 2) (Hou et al., 2022). Porphyrin photosensitizers co-assembled with copper-based agents include meso-tetra(4-aminophenyl) porphyrin (TAPP) and 5,10,15,20-tetrakis (4-carboxyphenyl) porphyrin (TCPP). In M-Cu-T, TAPP and Cu^2+^ synergistically generate massive ROS, causing mitochondrial dysfunction and activating the caspase-3/GSDME pathway to trigger tumor-specific immunogenic pyroptosis and initiate local anti-tumor immunity (Table 2) (Wang et al., 2023). TCPP in Cu@MIL-101@PMTPC produces ROS under 660 nm laser irradiation, cooperating with Fe^3+^ and surface Cu NPs to achieve enhanced PDT, effectively killing tumor cells and releasing DAMPs to mature DCs (Table 2) (An et al., 2024). To overcome limitations like poor water solubility, enzymatic degradation, aggregation, and self-quenching of porphyrins, MOF-based porphyrin photosensitizers have been developed. Cu-TCPP in UPIS acts both as a photosensitizer and a biomimetic nanozyme, enabling enhanced immunotherapy (Table 2) (Du J. et al., 2024). Similarly, porphyrin in Cu@COF-TATB not only serves as a photosensitizer for PDT-generated ^1^O_2_ but also provides binding sites for Cu^2+^ complexation. Cu@COF-TATB additionally catalyzes H_2_O_2_ into •OH via Fenton-like reactions. These multiplied ROS collectively induce tumor cell death and DAMP release, elevating immunogenicity and stimulating systemic anti-tumor immunity (Table 2) (Zhang et al., 2022).

**TABLE 2:** Multimodal immunotherapies of copper-based nanomedicine driven by external energy fields.

Formulations	Energy field	Responsive agent	Cu-based nanomedicine	Therapy	Cell type	Application	Ref.
CuET/ICG NPs	808 nm laser	ICG	Cu(DTC)_2_	PDT	4T1	Breast cancer	Zhang et al. (2025b)
HA@Cu(OH)_2_-ICG	808 nm laser	ICG	HA@Cu(OH)_2_	PDT	B16F10	Melanoma	Hou et al. (2022)
M-Cu-T	660 nm laser	TAPP	Cu-TAPP	PDT	LLC CT26 A549 H1299 PC9 H1975	Lung cancer Colon cancer	Wang et al. (2023)
Cu@MIL-101@PMTPC	660 nm laser	TCPP	Cu NPs	PDT	HepG2	Hepatocellular carcinoma	An et al. (2024)
UPIS	808 nm laser	TCPP	Cu-TCPP	PDT	4T1	Breast cancer	Du et al. (2024b)
3D Cu@COF-TATB	635 nm laser	TATB	Cu-TATB	PDT	4T1	Breast cancer anti-PD-L1 therapy	Zhang et al. (2022)
Cu-TBB	750 nm laser	TBB	Cu-TBB	PDT	4T1	Breast cancer	Zhang et al. (2023b)
PEG@Cu-AE NPs	450 nm laser	Aloe emodin	Cu-AE	PDT	LLC	Lung cancer anti-PD-L1 therapy	Yu et al. (2025)
(Cu_2_Se-CaO_2_)@LA	1,064 nm laser	Cu_2_Se	Cu_2_Se	PTT	4T1	Breast cancer	Feng et al. (2023a)
MACuS (η = 80%)	1,064 nm laser	MACuS	MACuS	PTT	4T1	Breast cancer	Zu et al. (2024)
HCuS-PE@TSL-tlyp-1	808 nm laser	HCuS NPs	HCuS NPs	PTT	4T1	Breast cancer	Wang et al. (2024b)
PEG@Cu_2_O-ES	1,064 nm laser	Cu_2_O-ES	Cu_2_O	PTT	4T1	Breast cancer anti-PD-1 therapy	Li et al. (2024a)
FG@Cu-CDs	808 nm laser	Cu-CDs	Cu-CDs	PTT	4T1	Breast cancer anti-PD-L1 therapy	Bao et al. (2023b)
CuSACO	1,064 nm laser	CuSA	CuSA	PTT	4T1 GL261	Breast cancer Glioma	Wu et al. (2024)
FeCu-DA	808 nm laser	FeCu-DA	FeCu-DA	PTT	4T1	Breast cancer anti-PD-L1 therapy	Ning et al. (2025)
Cu-IR783 NPs	Ultrasound	IR783	Cu-IR783 NPs	SDT	4T1 U87-MG	Glioblastoma	Hu et al. (2024a)
Cu/ART@Hpy	Ultrasound	Artesunate	Cu@Hpy	SDT	4T1	Breast cancer	Li et al. (2025a)
CMC	Ultrasound	Cu_2_O	Cu_2_O	SDT	4T1	Breast cancer	Cao et al. (2025)
ZCA NSs	Ultrasound	ZCA NSs	ZCA NSs	SDT	4T1 CT26	Breast cancer Colon cancer	Tang et al. (2024)
SCION-[Cu(DDC)_2_]	AMF	SCION	Cu(DDC)_2_	MHT	4T1 MDA-MB-231	Breast cancer	Cai et al. (2025)
Vk3@si@CuZnIONPs	AMF	CuZnIONPs	CuZnIONPs	MHT	A549	Lung cancer	Chauhan et al. (2023)
CSC@Syro	808 nm laser	CSC	CSC	PDT PTT	4T1	Breast cancer	Yang et al. (2023)
CMI-PEG	808 nm laser	CuMnO_x_ IR820	CuMnO_x_	PDT PTT	4T1	Breast cancer	Hu et al. (2024b)

Natural photosensitizers also show broad applicability. Cu-TBB releases Cu^+^ that catalyzes endogenous O_2_ to O_2_^−^•, which further undergoes SOD-catalyzed disproportionation and Haber-Weiss reactions to yield O_2_ and highly toxic •OH. Under 750 nm light, “switched-on” TBB simultaneously produces a ROS storm that activates GSDMD-mediated pyroptosis and promotes inflammatory cytokine release, enhancing DC maturation and T cell activation (Table 2) (Zhang Y. et al., 2023). Under 450 nm laser irradiation, Cu^2+^-loaded aloe emodin generates ROS, inducing ER stress and activating ICD for potent PDT. Intracellular Cu^2+^ accumulation disrupts the tricarboxylic acid cycle, eliciting proteotoxic stress and apoptosis, achieving synergistic immunotherapy (Table 2) (Yu et al., 2025).

#### 4.2.2 Photothermal copper-based nanomedicines

The application of PTT in multifunctional nanomaterials for enhancing Fenton-like reactions in tumor immunotherapy has garnered significant attention. On the one hand, PTT utilizes photothermal conversion to generate localized heating, ablating tumors and promoting the release of heat shock proteins. On the other hand, according to the Arrhenius equation, temperature elevation accelerates reaction kinetics, amplifying the ability of copper-based nanomedicines to regulate oxidative stress and activate tumor immunity. To date, various multifunctional copper-based nanomaterials, such as copper oxides, copper sulfides, copper selenides, copper single-atom materials, and copper-doped quantum dots, have been extensively studied for PTT in tumor immunotherapy, owing to their strong NIR absorption, high photothermal conversion efficiency (PCE), biocompatibility, and chemodynamic properties.

Copper selenides, including Cu_2_Se and CuSe, are attractive candidates for PTT. (Cu_2_Se-CaO_2_)@LA NPs exhibit a PCE of 51.6%. (Cu_2_Se-CaO_2_)@LA NPs exhibit a PCE of 51.6%. Upon NIR irradiation, the elevated temperature accelerates the Fenton-like reaction catalyzed by Cu_2_Se, enhancing ROS generation. This induces cancer cell death through mitochondria-associated ER stress, releasing DAMPs, with significantly superior efficacy compared to monotherapy with CDT or PTT alone (Table 2) (Feng X. et al., 2023). MACuS, assembled from copper sulfide (Cu_2-x_S), achieves a PCE of 80%. NIR-Ⅱ light not only directly activates MACuS for thermal tumor ablation but also accelerates the dynamic reaction between Cu^2+^/Cu^+^ and GSH within MACuS, leading to efficient apoptosis and cytotoxic ROS generation. Ultimately, immunogenic cuproptosis induced by MACuS activates systemic antitumor immunity, effectively inhibiting primary tumors as well as metastasis and recurrence in breast cancer models (Table 2) (Zu et al., 2024). HCuS-PE@TSL-tlyp-1 demonstrates a PCE of 22.69% under 808 nm irradiation. NIR irradiation triggers the dissolution of the thermosensitive lipid membrane (TSL-tlyp-1) via photothermal conversion, releasing piperazine-erastin and copper. The Cu^2+^/Cu^+^ species generate •OH via Fenton-like reactions, promoting lipid peroxide accumulation. Piperazine-erastin suppresses the antioxidant system by inhibiting the xCT pathway, synergistically achieving oxidative stress-mediated ferroptosis, releasing DAMPs to promote CTL infiltration and IFN-γ secretion (Table 2) (Wang et al., 2024b).

Cu_2_O in PEG@Cu_2_O-ES accelerates the release of ES and enhances Fenton-like reactions to generate massive ROS. The ROS attack ATP-dependent copper transporter, reducing Cu^2+^ efflux and intensifying cuproptosis, thereby enhancing sensitivity to anti-PD-1 therapy (Table 2) (Li W. et al., 2024). Copper-doped carbon dots (FG@Cu-CDs) exhibit a PCE of 35.13%. FG@Cu-CDs consume intracellular GSH and generate •OH, effectively remodeling the TIME (Table 2) (Bao et al., 2023b). Copper single-atom nanozymes CuSACO achieve a PCE of 46.9% under 1,064 nm laser irradiation. CuSACO synergistically activates tumor immunity through ROS storms, cuproptosis, and PTT, effectively suppressing the growth, recurrence, and metastasis of tumors (Table 2) (Wu et al., 2024). Similarly, dual-atom nanozymes FeCu-DA exhibit a PCE of 46.3%. PTT-enhanced cascade catalysis generates ROS, inducing potent cell death and DAMP release. FeCu-DA demonstrates synergistic therapeutic effects with anti-PD-L1 under NIR irradiation (Table 2) (Ning et al., 2025).

#### 4.2.3 Sonosensitive copper-based nanomedicines

Compared to PDT and PTT, sonodynamic therapy (SDT) offers deeper penetration, lacks phototoxicity, and has fewer side effects. It can induce the release of DAMPs and TAAs to promote immunotherapy without adversely affecting the immune system. Sonosensitizers are broadly categorized into organic and inorganic types. Notably, organic sonosensitizers can achieve stable, ultrasound-induced spatiotemporally controlled release through coordination co-assembly with Cu^2+^ or co-delivery. Among inorganic nanomaterials, some copper-based nanomedicines have shown potential as sonosensitizers. Furthermore, both organic and inorganic sonosensitizers benefit from the ability of Cu^+^/Cu^2+^ to trigger potent oxidative stress and induce RCD in tumor cells, expanding the application of copper-based nanomedicine in SDT.

IR783 typically fails to achieve *in situ* and visualized SDT due to its short circulation time and limited tumor accumulation. Benefiting from the EPR effect resulting from increased particle size upon assembly with Cu^2+^, Cu-IR783 NPs exhibit significantly higher tumor accumulation than free IR783. Cu-IR783 achieves deep tumor penetration and visualization *in situ* SDT through TME-responsive dissociation. Cu-IR783 NPs achieve SDT/cuproptosis by elevated ROS levels, inducing DAMPs release (Table 2) (Hu J. et al., 2024). Cu/ART@Hpy NPs, which coordinate Cu^2+^ and load artesunate, generate ROS under ultrasound to induce tumor cell apoptosis. They deplete intracellular GSH, enhancing Cu^2+^ accumulation to induce tumor cell cuproptosis, ultimately releasing DAMPs to activate tumor immunity (Table 2) (Li M. et al., 2025). Cu_2_O exhibits excellent sonodynamic and Fenton-like reaction activities due to its narrow bandgap and the presence of Cu^+^. Utilizing *in situ* generated Cu-MOF as a protective layer to load Cu_2_O, forming a Z-scheme heterojunction (CMC), results in superior sonodynamic activity. CMC elevates ROS levels, enhances cuproptosis, and releases DAMPs (Table 2) (Cao et al., 2025). Cu-substituted ZnAl ternary layered double hydroxide nanosheets (ZCA NSs) introduce Cu^2+^ into ZnAl nanosheets, inducing a strong Jahn-Teller effect accompanied by significant lattice distortion and atomic disorder. These abundant defects in ZCA NSs provide additional active sites for ROS generation and optimize the electronic structure, endowing ZCA NSs with excellent sonodynamic properties under ultrasound. Simultaneously, the released Cu^+^, synergistically enhanced by ultrasound irradiation, initiates SDT/cuproptosis through ROS, releasing DAMPs to promote DC maturation and elicit a systemic anti-tumor immune response (Table 2) (Tang et al., 2024).

#### 4.2.4 Magnetothermal copper-based nanomedicines

Due to the diamagnetic nature of biological tissues, magnetic hyperthermia therapy (MHT) offers deep tissue penetration, high site specificity, and minimal collateral damage. Similar to PTT, MHT enables remote, precise thermal control that can synergize with tumor immunotherapy to enhance Fenton-like reactions and activate antitumor immunity. Unlike PTT, MHT utilizes an alternating magnetic field (AMF) as its external stimulus.

The superparamagnetic copper iron oxide NP complex SCION-[Cu(DDC)_2_] generates localized heat under an AMF, promoting ROS production by Cu^2+^ and Fe^3+^ ions. The formation of the copper complex Cu(DDC)_2_ further amplifies oxidative stress, inducing ferroptosis and apoptosis while releasing DAMPs (Table 2) (Cai et al., 2025). Engineered Vk3@Si@CuZnIONPs possess multiple properties, including magnetization, •OH generation capability, and high heat production capacity. Doping copper and zinc into iron oxide NPs endows them with high heating efficiency. Under an AMF, Vk3@Si@CuZnIONPs efficiently generate endogenous heat, enhancing the efficiency of ROS generation by Cu^2+^ and Zn^2+^ ions while sensitizing DNA to ROS damage. The magnetothermal effect combined with massive ROS further induces tumor cell apoptosis, upregulates heat shock protein and pro-inflammatory cytokine expression, and ultimately activates immune responses (Table 2) (Chauhan et al., 2023).

#### 4.2.5 Multifunctional copper-based nanomedicines

Recent research has focused on multimodal tumor immunotherapy utilizing multifunctional copper-based nanomaterials. Representative examples include the combination of PDT/PTT and PDT/SDT with oxidative stress-based therapies. CuSe/CoSe_2_@syrosingopine not only reverses the immunosuppressive TME via lactate reprogramming but also triggers potent antitumor immunity through synergistic ferroptosis, PTT, and PDT (Table 2) (Yang et al., 2023). CMI-PEG, composed of porous CuMnOx loaded with IR820 and surface-PEGylated, employs synergistic PTT/PDT. It enhances oxygen generation and depletes GSH, thereby inducing robust oxidative stress to effectively promote tumor cell apoptosis and DAMP release. This stimulates T cell infiltration and augments antitumor immune responses (Table 2) (Hu P. et al., 2024). Furthermore, combining copper-based nanomedicines with microwave sensitizers leverages microwave irradiation to facilitate electron transitions. This approach converts microwave energy into heat and promotes ROS generation, unimpeded by gas or bone interference. These advantages endow copper-based agents with the potential to enhance tumor immunotherapy by regulating oxidative stress (Feng Y. et al., 2023). Despite the enhanced antitumor efficacy achieved through various combination strategies of copper-based nanomedicines, their clinical translation faces critical technical challenges. A prominent issue is the inherent complexity of combination approaches. Most sensitizers feature intricate structures lacking sufficient biocompatibility and biodegradability, severely impeding large-scale production and practical application. Secondly, external energy fields risk off-target effects, such as thermal damage to normal tissues, necessitating the design of highly specific nanomedicines to minimize treatment-induced side effects. Current multimodal strategies predominantly utilize subcutaneous tumor models in mice, overlooking the anatomical positioning and complexity of orthotopic tumors, which significantly diminishes their translational potential for human applications. For instance, SDT- and PDT-dependent equipment requires substantial optimization to address practical disparities between murine models and human physiology. Concurrently, advanced integrated devices enabling multimodal therapeutic combinations must be developed to ensure clinical feasibility and utility. Collectively, these multifunctional combinations represent a transformative advancement in copper-based nanomedicine-regulated oxidative stress for activating tumor immunity.

## 5 Conclusion and future perspectives

The critical role of metal ions in the TIME has established metalloimmunotherapy as a pivotal concept. Recent advancements in nanotechnology have significantly propelled the development of this field. Copper, as an essential transition metal for human immune regulation, deserves special attention in metalloimmunotherapy. Given tumor cells’ higher redox potential compared to normal cells, oxidative stress modulation serves as the core mechanism for copper-based nanomedicines to activate tumor immunity. Unlike other metal ions, Cu^+^/Cu^2+^ catalyzes more potent Fenton-like reactions for ROS generation, demonstrating exceptional performance in activating antitumor immunity. This review comprehensively summarizes the role of copper-based nanomedicines in modulating oxidative stress for tumor immune activation, including inducing various forms of RCD to restore tumor immunogenicity and establish crosstalk with ICD, activating immune cells to orchestrate innate and adaptive immunity, and reprogramming small-molecule metabolites to reverse immunosuppressive microenvironments. These mechanisms highlight the immense potential of oxidative stress regulation in copper-based tumor immunotherapy. As an effective strategy, copper-based nanomedicines synergize with multiple therapeutic approaches to elicit stronger antitumor immune responses than oxidative stress monotherapy. The systematic integration of these strategies provides critical insights for designing multifunctional nanotherapeutic platforms centered on copper-based agents and oxidative stress, while offering guidance for developing next-generation copper-based nanomedicines to enhance anti-tumor immunity.

Currently, fundamental research evidence supporting the reinforcement of anti-tumor immunity through oxidative stress modulated by copper-based nanomedicines is continuously emerging. However, before this new advancement in copper-based nanomedicines for tumor immunotherapy moves to the next stage, several issues and challenges require close attention and careful resolution. To begin with, the oxidative stress modulated by copper-based nanomedicines must be finely controlled. Copper-based nanomedicines, possessing strong Fenton-like reactivity, offer a broader range for precisely tuning ROS generation. Most studies tend to induce a powerful ROS storm to activate tumor immunity. However, it is worth emphasizing that the role of ROS in the TIME is complex and dual-faceted. Specifically, ROS can not only activate anti-tumor immunity but also promote immune escape and tumor metastasis. Uncontrolled, excessive ROS may have detrimental effects on the TIME. The induction of a ROS storm in tumor immunotherapy cannot be viewed as a one-size-fits-all modality. The heterogeneity of the tumor redox state requires careful consideration. Correlating clinical parameters, mutational profiles, transcriptomics, metallomics, proteomics, and metabolomics features with the tumor redox status will help identify cancer patient subpopulations who may potentially benefit from copper-based nanomedicines.

The structure-activity relationships of copper-based nanomedicines deserve significant attention. Although theoretical calculations, such as Density Functional Theory and molecular dynamics simulations are crucial for understanding the physicochemical properties of copper-based nanomedicines. The spatiotemporal complexity of the *in vivo* biological environment makes it difficult to precisely delineate their specific mechanisms of action, particularly concerning their impact on immune cells and immune-related pathways. Further research is required to explore the crosstalk mechanisms between copper-based nanomedicines and immune cells, particularly with respect to T-cell exhaustion, TAM polarization, and the cGAS-STING pathway. Beyond microscopic-level regulation, such as metal valence state and coordination environment, research on the macroscopic level should also focus on how material characteristics, including morphology, structure, size, surface modification, and formulation, affect oxidative stress generation, mechanism of action, and pharmacokinetics (ADMET: Absorption, Distribution, Metabolism, Excretion, Toxicity). Long-term exposure to metal ions may lead to chronic toxicity, immune system disorders, or endocrine disruption. Given that immunotherapy typically requires repeated administration, the long-term toxicity of copper-based nanomedicines warrant significant attention. Copper ions exhibit cytotoxicity at high concentrations and can cause non-specific cellular damage. Copper-based nanomedicines may accumulate in non-target organs such as the liver and kidneys, potentially resulting in chronic toxicity and organ damage. The accumulation of nanomedicines in the liver and kidneys is essentially attributed to their size-dependent clearance efficiency. Oversized nanomedicines are trapped by physical/immune barriers (e.g., phagocytosis by hepatic macrophages or mechanical obstruction in the glomeruli), while undersized nanomedicines are constrained by biointerface interactions (e.g., renal tubular reabsorption or sub-nanoscale adhesion). To enhance the immune response after administration of copper-based nanomedicines, a rational size design is critical. Generally, a size range of 10–100 μm is optimal to enhance lymph node drainage and suppress systemic diffusion into the bloodstream. Notably, although smaller nanoparticles can efficiently penetrate tumors, larger nanoparticles may be more advantageous for targeting tumor-associated macrophages. Future studies must focus on systematically investigating the chronic toxicity of copper-based nanomedicines and other metal-based nanomedicines. In tumor immunotherapy, rational surface modification and precise control of the size of copper-based nanomedicines are essential to ensure their specific accumulation and efficacy at tumor sites. Recent progress in machine learning for nanomaterial screening offers new insights for novel nanomedicine research, including in areas such as surface adsorption, supramolecular recognition, surface catalysis, and chemical transformation. Machine learning presents new opportunities for copper-based nanomedicines to modulate oxidative stress and activate tumor immunity, potentially significantly reducing the costs associated with traditional theoretical calculations and experimental trial-and-error. However, given the complexity of nanomaterials and their interactions with the biological immune system, constructing high-quality datasets, including datasets for copper-based nanomedicine development and multi-omics datasets focused on mechanisms of action, is essential for realizing an integrated approach of “computation + machine learning + experiment.”

In terms of the synthesis of copper-based nanomedicines, most originate from derivatives of traditional industrial catalysts, lacking design and consideration from a biological perspective. Improvements in preparation methods and the development of controllable, stable production processes could facilitate further research and application of copper-based nanomedicines. Furthermore, biomimetic copper-based nanomedicines, such as single-atom structures, are constructed using carbon-based precursors processed in an inert atmosphere at high temperatures (above 850 °C). Due to the high degree of carbonization in the matrix, the solubility of the carbon substrate is poor, which can impair its ability to generate oxidative stress. While simple surface modifications may improve its targeting and bioavailability, employing sophisticated carriers, such as liposomes and exosomes, could lead to new breakthroughs. Simultaneously, the catalytic action of copper-based nanomedicines may exhibit low selectivity within the complex TIME, leading to uncontrolled side reactions. Adjusting the electronic structure of the catalytic center, spatial confinement, and inspiration from enzyme-ligand catalytic systems could help enhance the selectivity of oxidative stress modulation by copper-based nanomedicines.

Although multimodal tumor immunotherapy based on copper-based nanomedicine-regulated oxidative stress overcomes the limitations of single therapeutic modalities, the complexity of some multimodal approaches makes precise mechanistic elucidation and practical application difficult. Examples include the co-delivery of multiple drugs and the activation of multiple energy fields. Carefully studying the efficacy and complexity of different strategies will aid in developing more economical and practical copper-based nanomedicines for inducing oxidative stress to achieve tumor immunotherapy. Regarding co-delivery strategies, the intricate synergistic effects between copper and other metals on the TIME require further clarification. Currently, although various co-delivery combinations of drugs with copper-based nanomedicines have been developed, these represent only the tip of the iceberg of tumor immunotherapeutic agents. More potential candidates could be sourced from small-molecule inhibitors, repurposed drugs, and natural products. Microorganisms possess metabolic patterns and redox states distinct from human cells; incorporating microbiota into co-delivery components could have profound implications for activating tumor immunity via copper-based nanomedicine-regulated oxidative stress. Developing and designing specific co-delivery components, such as antibodies, nucleic acids, and proteins/peptides, is beneficial for unlocking the full potential of copper-based nanomedicines in tumor immunotherapy.

In conclusion, copper-based nanomedicines demonstrate intricate mechanisms in regulating oxidative stress for tumor immune activation, with profound implications spanning tumor cell fate determination, immune cell stimulation, and small-molecule metabolism modulation. Advances in mechanistic understanding have enabled effective multimodal immunotherapy strategies leveraging oxidative stress modulated by copper-based nanomedicine. As a novel, safe, and effective immunotherapeutic platform, copper-based nanomedicines hold significant clinical promise. Nevertheless, substantial challenges persist in their application for tumor immunity through oxidative stress modulation, necessitating in-depth investigation into action mechanisms, structure-activity relationships, precision design, batch synthesis, catalytic selectivity, multimodal therapeutic integration, and co-delivery strategies. Our review offers valuable insights for advancing copper-based nanomedicine immunotherapy and redox-based immunotherapy, while providing novel perspectives for developing effective cancer immunotherapies.

## Glossary

AMF: alternating magnetic field
ATP: adenosine triphosphate
CAT: catalase
CDT: chemodynamic therapy
CTL: cytotoxic T lymphocytes
DAMP: damage-associated molecular pattern
DC: dendritic cells
DLAT: dihydrollipoamide S-acetyltransferase
DSF: disulfiram
DOX: doxorubicin
ES: elesclomol
ER: endoplasmic reticulum
EPR: enhanced permeability and retention
FDX1: ferredoxin 1
GSDM: gasdermin
GOx: glucose oxidase
GSH: glutathione
GPX4: glutathione peroxidase 4
HMGB1: high mobility group box 1
HSP: heat shock protein
ICD: immunogenic cell death
ICG: indocyanine green
LIAS: lipoic acid synthetase
Lox: lactate oxidase
MHT: magnetic hyperthermia therapy
MTO: mitoxantrone
NP: nanoparticle
NIR: near-infrared
OXD: oxidase
PCE: photothermal conversion efficiency
PDT: photodynamic therapy
PD-L1: programmed death-ligand 1
PD-1: programmed cell death protein 1
POD: peroxidase
PTT: photothermal therapy
ROS: reactive oxygen species
RCD: regulated cell death
SDT: sonodynamic therapy
SOD: superoxide dismutase
TLR: Toll-like receptor
TAA: tumor-associated antigen
TAM: tumor-associated macrophage
TIME: tumor immune microenvironment
TME: tumor microenvironment.
